# Non-conventional Ultra-High Dose Rate (FLASH) Microbeam Radiotherapy Provides Superior Normal Tissue Sparing in Rat Lung Compared to Non-conventional Ultra-High Dose Rate (FLASH) Radiotherapy

**DOI:** 10.7759/cureus.19317

**Published:** 2021-11-06

**Authors:** Michael D Wright, Pantaleo Romanelli, Alberto Bravin, Geraldine Le Duc, Elke Brauer-Krisch, Herwig Requardt, Stefan Bartzsch, Ruslan Hlushchuk, Jean-Albert Laissue, Valentin Djonov

**Affiliations:** 1 Ginzton Technology Center, Varian Medical Systems, Palo Alto, USA; 2 Research & Development Center, Avail Medical Devices, Roseville, USA; 3 Cyberknife Center, Centro Diagnostico Italiano, Milano, ITA; 4 Biomedical Beamline, European Synchrotron Radiation Facility, Grenoble, FRA; 5 Pharmaceutics, NH TherAguix, Lyon, FRA; 6 Department of Radiation Oncology, School of Medicine, Technical University of Munich, Munich, DEU; 7 Institute for Radiation Medicine, Helmholtz Centre Munich, Munich, DEU; 8 Institute of Anatomy, University of Bern, Bern, CHE

**Keywords:** microbeam(s), flash, radiotherapy, lung cancer, fibrosis

## Abstract

Conventional radiotherapy is a widely used non-invasive form of treatment for many types of cancer. However, due to a low threshold in the lung for radiation-induced normal tissue damage, it is of less utility in treating lung cancer. For this reason, surgery is the preferred treatment for lung cancer, which has the detriment of being highly invasive. Non-conventional ultra-high dose rate (FLASH) radiotherapy is currently of great interest in the radiotherapy community due to demonstrations of reduced normal tissue toxicity in lung and other anatomy. This study investigates the effects of FLASH microbeam radiotherapy, which in addition to ultra-high dose rate incorporates a spatial segmentation of the radiation field, on the normal lung tissue of rats. With a focus on fibrotic damage, this work demonstrates that FLASH microbeam radiotherapy provides an order of magnitude increase in normal tissue radio-resistance compared to FLASH radiotherapy. This result suggests FLASH microbeam radiotherapy holds promise for much improved non-invasive control of lung cancer.

## Introduction

Every year there are 2.2 million new cases of lung cancer globally and 1.8 million lung cancer deaths. Lung cancer accounts for 11% of all new cancer reports, and 18% of all cancer deaths. It is the leading form of cancer-related death. The five-year survival rate for new lung cancer patients is 10-20% [1,2]. Lung cancer is clearly a deadly disease of a vast proportion.

The standard of care for lung cancer is surgery [3,4]. For early-stage tumors, most often an entire lobe of the lung is removed. Such surgery is extremely invasive, requiring weeks to months of recovery time.

Conventional radiotherapy (CONV-RT) is a noninvasive form of cancer therapy that works well for many forms of cancer. For lung cancer, however, CONV-RT is generally reserved for cases where resection is not possible or for late-stage malignancies where multi-mode treatment is required [3,4]. The reason is normal tissue toxicity.

The lung is among the most radiosensitive of organs, and radiation-induced injury is common [5-8]. Pneumonitis, a type of inflammation, is an acute injury that generally presents within the first six months following treatment [6,8]. Pulmonary fibrosis is a late-stage injury that typically manifests in the time period from six to 24 months post-irradiation [6,8]. Pneumonitis is often resolved with steroid medications, while currently there is no good therapeutic intervention for fibrosis available [6,7]. Pulmonary fibrosis is characterized by the destruction of lung parenchyma and the generation of fibrotic tissue which reduces gas exchange between blood cells and air and stiffens the lung structure making it more difficult to expand the lungs. These effects may combine to produce several severe symptoms such as chronic shortness of breath, fatigue, weakness, chest pain, and, potentially, death. The injury is both progressive and irreversible [7]. A radiological study of lung damage post-conventional stereotactic body radiation therapy showed radiological abnormalities in 54% of patients within six months of irradiation, and in 99% of patients after thirty-six months [9]. Such prevalent signs of normal tissue toxicity severely restrict the use of CONV-RT for lung cancer.

The concept of ultra-high dose rate radiotherapy (FLASH-RT) was introduced by Favaudon et al. [10], spawning much interest in the radiotherapy community [11-15]. The Favaudon et al. study investigated the effects of conventional and FLASH dose rates on both normal and tumoral tissue in the lungs of mice. At a single fraction dose of 15 Gy, both the conventional dose rate (~ 0.03 Gy/s) and the ultra-high dose rate (~ 60 Gy/s) radiation resulted in equivalent tumor control. However, at a single-fraction dose of 17 Gy, the mice subjected to the conventional dose rate exhibited severe lung fibrosis, while the mice subjected to the ultra-high dose rate exhibited only low-grade fibrosis. A dose escalation to 30 Gy was required to induce equally severe lung fibrosis in the ultra-high dose rate case.

Ultra-high dose rate microbeam radiotherapy (FLASH-MRT) presents a potential improvement to FLASH-RT by adding a spatial fractionation component to the radiation while maintaining the temporal component. In FLASH-MRT, the radiation field is segmented into very thin planes of radiation separated by spaces. The planes of radiation are from a few tens to a few hundreds of microns wide. The spaces are from a few hundred microns to a few millimeters wide. This contrasts with both CONV-RT and FLASH-RT in which the radiation field is continuous across the target, i.e., broad beam.

Studies of the effect of FLASH-MRT on brain tumors in small animals have demonstrated that tumors can be controlled with *no* normal tissue toxicity, i.e., no apparent loss of normal tissue functionality [16-19]. These studies have also established that normal brain tissue can tolerate enormous doses before there is evidence of irreparable damage. In CONV-RT, the cumulative dose delivered to most gliomas is kept below 60 Gy in order to avoid normal tissue toxicity, whereas in FLASH-MRT peak doses of several hundreds of Gy have been tolerated by normal brain tissue [16-23].

This article presents a first pre-clinical study to determine whether FLASH-MRT may be effective in the fight against lung cancer. The study examines the effects of both FLASH-MRT and FLASH-RT on the normal lung tissue of rats, with fibrotic damage as the focus of histopathological examination.

## Materials and methods

Overview

The microbeam irradiations were performed at the Biomedical Beamline of the European Synchrotron Radiation Facility (ESRF) in Grenoble, France [24]. The Fischer-344 male rat (Charles River Laboratories, L’-Arbresle, France) was chosen as the animal model for the experiment with thirty-three animals utilized in total. These animals were randomly distributed among eleven groups, two to four animals per group. Three radiation field patterns were employed: broad beam, microbeams 50$\mu$m wide on a 400$\mu$m pitch, and microbeams 500$\mu$m wide on a 4 mm pitch. One group of animals was designated as the control group and not exposed to radiation. Two groups of animals were exposed to broad beam radiation. An entrance dose of 30 Gy was delivered to the first of these groups, and 50 Gy to the second. Four groups of animals were exposed to the 50$\mu$m wide, 400$\mu$m pitch microbeams, with peak entrance doses of 50, 100, 300, and 600 Gy. Similarly, four groups of animals were exposed to the 500$\mu$m wide, 4 mm pitch microbeams with peak entrance doses of 50, 100, 300, and 600 Gy.

For each animal exposed to radiation, the radiation was delivered to the right lung in a 1 cm x 1 cm window. Image guidance was used to avoid the spinal cord, the heart, and the liver. For the 50$\mu$m wide microbeams on a 400$\mu$m pitch, a total of twenty-five microbeams were contained in the 1 cm x 1 cm window. For the 500$\mu$m wide microbeams on a 4 mm pitch, three microbeams were contained in the window. A respirator was used to arrest lung motion during irradiation. In all cases, the radiation dose was delivered in a single fraction at an ultra-high dose rate. All animals were sacrificed for histopathological analysis at 12 months post-irradiation. Pathologists scoring tissue samples were blinded to the radiation field patterns and doses applied.

Irradiation

Animals were irradiated by moving them vertically through a stationary radiation field. At the target plane, the maximum possible radiation field was a broad beam 40 mm horizontal x 0.5 mm vertical in size. The horizontal beam divergence was 1 mrad and the vertical beam divergence was 0.02 mrad. Remotely controlled horizontal and vertical tungsten slits were used to perform the first collimation of the beam envelope at the target. Custom-made multi-vertical slit tungsten collimators were additionally used to pattern the radiation field into arrays of microbeams [25].

**Figure 1:**
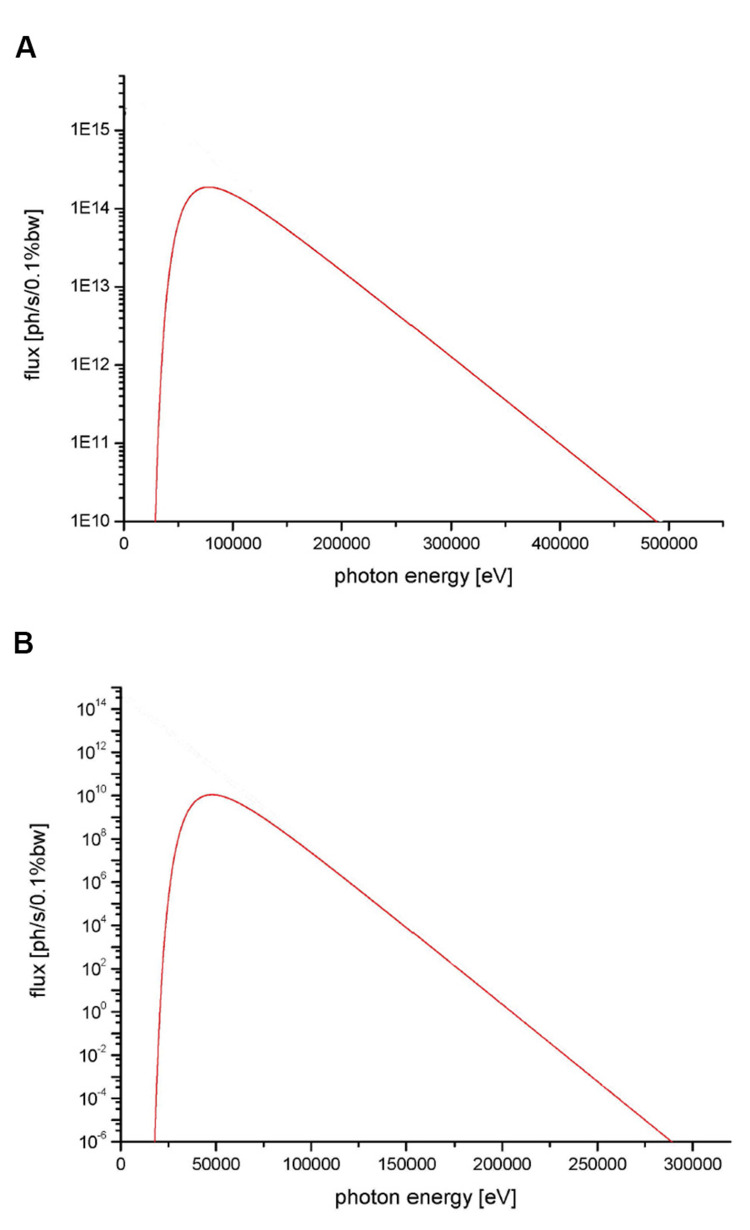
X-ray spectra. (A) Therapeutic spectra. (B) Imaging spectra.

Two X-ray spectra were used, one for therapeutic-level radiation and one for imaging. The therapeutic-level spectrum had a peak energy of 81 keV and a mean energy of 107 keV. The imaging spectrum had peak energy of 48 keV and a mean energy of 60 keV. See Figure 1.

Radiation was delivered to the target specimen in pulses. The temporal width of each pulse was 2.3 x 10^-11^ s, and the pulse frequency was 3.52 x 10^8^ Hz. For the therapeutic-level radiation, each pulse provided 3.98 x 10^-5^ Gy. Hence, the instantaneous therapeutic-level dose rate associated with each pulse was 1.73 x 10^6^ Gy/s, and the average dose rate was 1.4 x 10^4^ Gy/s. The corresponding dose rates for imaging radiation were four orders of magnitude lower. For imaging, the multi-slit collimator was removed from the beam path.

To assure proper targeting of the therapeutic-level radiation, the subject animal was imaged first, thereby providing image guidance. The imaging system consisted of a fast readout, low noise (FreLoN) 2048 x 2048 charge-coupled device (CCD) camera coupled with visible light magnifying lenses located 3 m downstream of the animal [26]. The x-rays were converted into green light by a Gd_2_O_2_S fluorescent screen placed at the entrance of the optics. The effective pixel size was 23.3$\mu$m at the target plane [27]. The entire thorax of the animal was imaged, requiring a 9 s scan through the imaging radiation field. The imaging dose to the animal was 350 mGy. The image was used to locate, with better than 100$\mu$m precision, the 1 cm x 1 cm window for therapeutic-level radiation.

The vertical scan speed of the animal through the stationary radiation field was determined by the following formula:

$v= \frac{z}{\left ( \frac{D}{\gamma } \right )}$

where $v=$ vertical scan speed, $z=$ radiation field height, $D=$ target dose, and $\gamma =$ average dose rate.

For therapeutic-level radiation, two values of z were used, 0.05 and 0.5 mm. The smaller value was chosen for the lower target doses of 30 and 50 Gy, and the larger value for the higher target doses of 100, 300, and 600 Gy. The highest vertical scan speed was required to deliver 100 Gy to the target, equaling 8 cm/s. The lowest vertical scan speed was required to deliver 600 Gy to the target, equaling 1.3 cm/s.

For the broad beam radiation pattern, the radiation filled 100% of the 1 cm x 1 cm window. For the radiation pattern comprised of 50$\mu$m wide microbeams on a 400$\mu$m pitch, the sum of all the 50$\mu$m radiation peaks occupied 13% of the radiation window. For the radiation pattern comprised of 500$\mu$m wide microbeams on a 4 mm pitch, the sum of all the 500$\mu$m radiation peaks occupied 15% of the radiation window.

Animal handling

All procedures related to animal care conformed to the guidelines of the French government (licenses 380325 and B3818510002) and were approved by the ESRF Internal Evaluation Committee for animal welfare and rights.

The animals were maintained under controlled environmental conditions (22$\pm$2 ^o^C, 40-60% humidity, and a 12 hour light/dark cycle), with food and water ad libitum. All efforts were made to minimize the potential suffering and discomfort of the animals. Animals were checked daily and weighed weekly.

To arrest lung motion during irradiation, thereby avoiding the widening of radiation damage tracks created by microbeams, the animals were subjected to controlled breathing via an Alphalab respirator (Minerve Equipment Vétérinaire, Esternay, France). In preparation for connection to the respirator, the subject animal was anesthetized with isoflurane gas (5% for two minutes, then 1.5% for maintenance) and an intraperitoneal injection of a xylazine/ketamine cocktail (64.5/5.4 mg/kg). A subcutaneous injection of atropine (0.1 mg/ml, 1 ml/kg) was also made to avoid diaphragm spasms and to block mucus secretion at the trachea. The animal was then mounted vertically on a plastic support frame and fixed by the teeth. The throat and trachea were coated with lidocaine, a local anesthetic, to prevent the reflexive closure of the vocal cords during intubation. The animal was intubated with the aid of a laryngoscope. Once the endotracheal tube was inserted, it was then connected to the respirator operating in spontaneous mode. The animal was then installed on a Kappa-type goniometer (Huber, Rimsting, Germany) in the experimental hutch, oriented such that the anterior of the thorax faced the beam, and the nose of the animal pointed vertically up.

In the period prior to the delivery of imaging or therapeutic-level radiation, the respirator was operated in the assisted mode set at 80 breathing cycles/min, each cycle comprised of one inspiration followed by two expirations. The gas mixture during this mode was 1.5% isoflurane and 98.5% air. Two minutes prior to radiation delivery, the animal was hyperventilated by changing the gas mixture to 3% isoflurane and 97% pure O2. The total gas flow was set at 0.2 l/min.

Immediately following hyperventilation, the animal was placed into apnea by insufflating an overpressure in the animal’s lungs. This was accomplished using the hold-pressure mode of the respirator. The animal was capable of remaining in apnea for up to 1 min before an inspiration reflex. Typically, the animal remained in apnea less than 30 s for the delivery of either imaging or therapeutic-level radiation.

For the animals exposed to broad beam therapeutic-level radiation, it was only necessary to induce apnea during imaging. During the delivery of therapeutic-level radiation, these animals were allowed to breathe in spontaneous mode.

At 12 months post-irradiation, all animals were sacrificed for histopathological analysis. This was accomplished by first anesthetizing with a gas mixture of 5% isoflurane and 95% air, followed by intraperitoneal injection of a lethal dose of pentobarbital.

Dosimetry

The establishment of synchrotron radiation beam parameters necessary to deliver the desired microbeam peak doses was a three-step process. In the first step, dose measurements for a broad beam radiation field were performed under predefined reference conditions, which correlated machine parameters such as synchrotron storage ring current with reference dose rate. In the second step, Monte Carlo calculations modeled these reference conditions and provided a calibration between broad beam measurements and Monte Carlo broad beam simulations. In the third step, Monte Carlo simulations determined absorbed dose maps of the collimator-shaped microbeams in the target volume and hence established a link between microbeam peak dose and broad beam dose. 

Broad beam dose measurements were made in compliance with the recommendations of the International Atomic Energy Agency Technical Report Series No. 398 [28]. The measurements were made at reference conditions in a water phantom at a depth of 2 cm for a 2 cm x 2 cm radiation field. A pinpoint ionization chamber of cylindrical shape with a volume of 0.015 cm^3^ (Model 31014, PTW, Freiburg, Germany) was used, connected with a Unidos electrometer (PTW, Freiburg, Germany). The ionization chamber was scanned through the radiation field at a speed of 2 cm/s. 

Monte Carlo simulations were performed using the Geant4 toolkit version 10.03 [29]. For predictions of depth-dose values, the definition and shape of the phantom in these simulations were based on Hounsfield units from the CT of a rat, which was acquired at the ESRF with a narrow bandwidth photon beam peaking at 35 keV and a resolution of 33 µm. The Hounsfield units were converted into material composition and density using the interpolation method of Schneider et al. [30]. Peak and valley doses were scored on the grid predefined by the CT cube [31]. A total number of 10^9^ photon histories were simulated. For predictions of the lateral-dose profiles of the microbeam arrays, a water phantom was utilized with dose scored on a mesh with 5$\mu$m pitch perpendicular to the beams. A total of 2 x 10^9^ photon histories were simulated. For all simulations, production cut-offs for electrons and photons were set to 1$\mu$m. Valley dose corresponds to the average dose in the central 60% of the valley, and peak dose is the average dose in the central 80% of the peak. The model of the source incorporated beam spectrum, polarization, collimator absorption, and leakage radiation [32]. The combination of reference dosimetry, Monte Carlo simulations at reference conditions, and Monte Carlo simulations of the experimental set-up with microbeams allowed the prediction of absolute dose values.

Histology

The lungs and heart of each animal in the study were harvested as a single block of tissue. The lungs were fixed by immersion into 4% paraformaldehyde (PAF) in phosphate-buffered saline (PBS). The tissue block was then embedded in paraffin and sliced into 5$\mu$m thick sections for histological study.

Coronal sections of the right lung, usually including parts of the upper, middle, and lower right lobes, and coronal sections of the left lung, were taken at an anteroposterior tissue depth of approximately 2 cm. Sections were also taken from all sites that displayed macroscopic changes.

The scheme of Hübner et al. [33] used to grade bleomycin-induced pulmonary fibrosis in rats was adapted for the scoring of microbeam radiation-induced fibrosis in this study. In the Hübner work, the administration of bleomycin by intratracheal injection and subsequent air injections produced widespread heterogeneous fibrotic damage throughout the lungs. The Hübner grading scheme accounted for both the changes in lung parenchyma and the amount of lung area displaying such changes. The modified scores used in this study follow precisely the Hübner descriptions of lung parenchyma damage. See Table 1. However, because microbeams traverse quite limited areas of the lung, the area component of the Hübner grading scheme was not appropriate for this study. Instead, the areas with a damage score of three and higher in selected animals were measured and recorded as a percentage of the radiation window area. As a benchmark, it is to be noted that in the Hübner work, a grade of five, consisting of confluent fibrotic masses occupying between 10 and 50% of the lung sample area, represented severe lung damage.

**Table 1:** Modified Hübner scoring scheme for fibrosis.

Fibrosis Score	Description
0	Alveolar septa: No fibrotic damage. Lung structure: Normal lung.
1	Alveolar septa: Isolated gentle fibrotic changes (septum $\leq$ 3x thicker than normal). Lung structure: Alveoli partly enlarged and rarefied, but no fibrotic masses present.
2	Alveolar septa: Clearly fibrotic changes (septum $>$ 3x thicker than normal) with knot-like formation but not connected to each other. Lung structure: Alveoli partly enlarged and rarefied, but no fibrotic masses present.
3	Alveolar septa: Contiguous fibrotic walls (septum $>$ 3x thicker than normal). Lung structure: Alveoli partly enlarged and rarefied, but no fibrotic masses present.
4	Alveolar septa: Variable. Lung structure: Single fibrotic masses.
5	Alveolar septa: Variable. Lung structure: Confluent fibrotic masses. Lung architecture severely damaged but still preserved.
6	Alveolar septa: Variable, mostly nonexistent. Lung structure: Large contiguous fibrotic masses. Lung architecture mostly not preserved.
7	Alveolar septa: Nonexistent. Lung structure: Alveoli nearly obliterated with fibrous masses.
8	Alveolar septa: Nonexistent. Lung structure: Complete obliteration with fibrotic masses.

Masson-Trichrome stained tissue sections selected for inspection were scanned in a raster-like pattern using an Axiophot microscope (Zeiss AG, Feldbach, Switzerland) with a 20x objective. All areas displaying fibrosis were measured, scored, and photographed.

For histopathological analysis, standardized international guidelines for diagnostic criteria for lesions of the respiratory tract in rats and mice were followed [34]. Evaluation of tissue sections was conducted with the scorers blinded to the radiation field patterns and doses applied.

## Results

Depth-dose values

Monte Carlo calculations employed in this study predict the peak and valley doses as a function of depth into an animal for each of the radiation field profiles. Table 2 shows these peak and valley doses at a depth of 2 cm, the depth into an animal at which tissue samples were acquired for histopathological analysis.

**Table 2:** Calculated depth-dose values. Doses at peak and valley centers at 2 cm depth into tissue as a function of peak entrance dose. All depth-dose values are in Gy. Values have an uncertainty of approximately ± 5%.

Radiation Field Pattern	Location	Depth-Dose Value				
		Peak Entrance Dose				
		30 Gy	50 Gy	100 Gy	300 Gy	600 Gy
Broad beam	Peak	24	41	-	-	-
	Valley	-	-	-	-	-
Microbeams 50 $\mu$m wide, 400 $\mu$m pitch	Peak	-	41	81	244	488
	Valley	-	0.99	1.98	5.94	11.9
Microbeams 500 $\mu$m wide, 4 mm pitch	Peak	-	42	85	254	509
	Valley	-	0.56	1.12	3.36	6.72

Lateral-dose profiles

**Figure 2:**
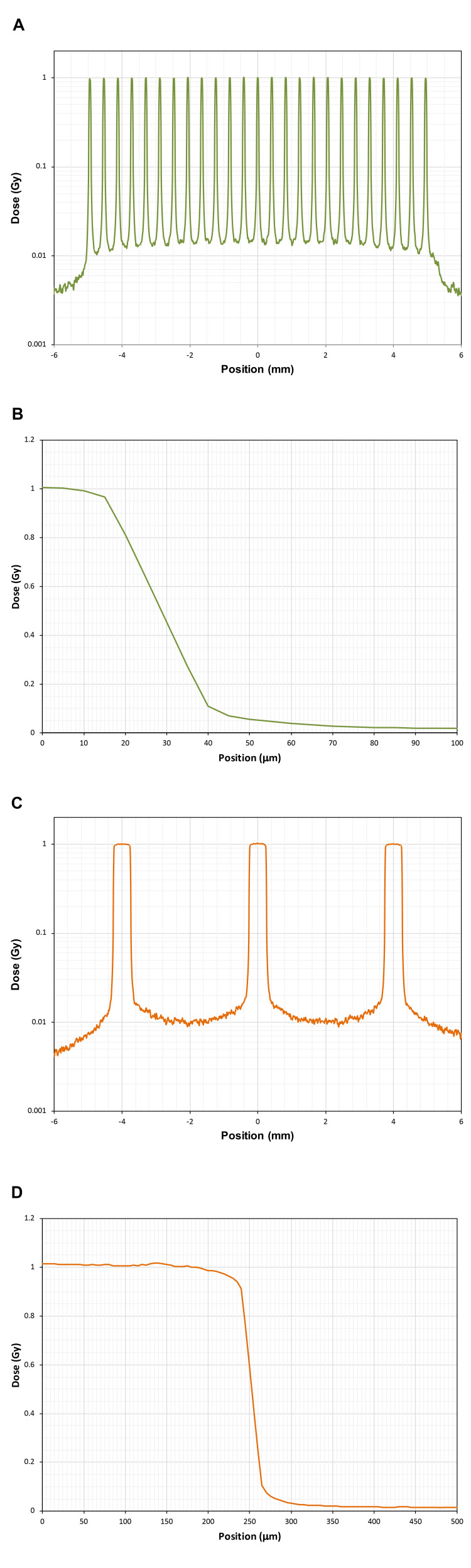
Lateral-dose profiles. All dose maps are normalized to a peak dose of 1 Gy. (A) Dose map for the array of 50$\mu$m wide microbeams on a 400$\mu$m pitch, logarithmic scale. (B) Dose map for a single 50$\mu$m wide microbeam, extending from the center of the peak, linear scale. (C) Dose map for the array of 500$\mu$m wide microbeams on a 4 mm pitch, logarithmic scale. (D) Dose map for a single 500$\mu$m wide microbeam extending from the center of the peak, linear scale.

Monte Carlo predictions of the lateral-dose maps for the microbeam radiation fields are shown in Figure 2. These dose profiles are calculated for a depth of 3 mm in water. The 90%-to-10% penumbra widths are approximately 25$\mu$m for both the 50$\mu$m and 500$\mu$m wide microbeam arrays.

Macroscopy

**Figure 3:**
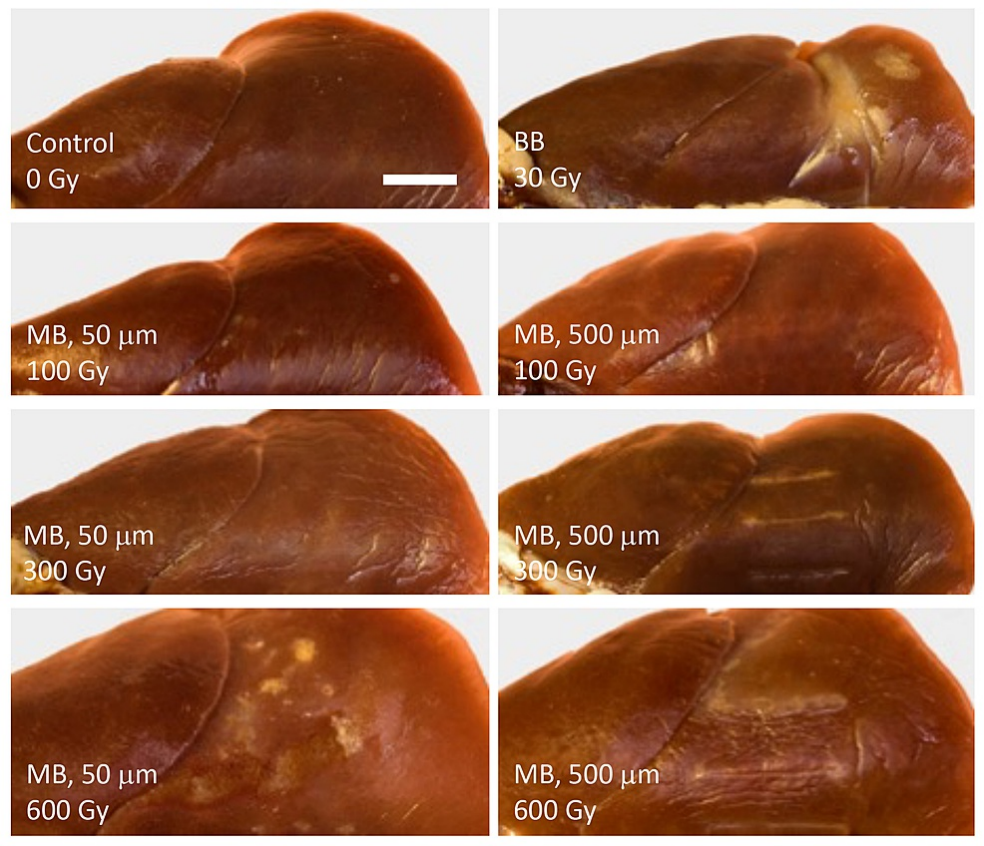
Macroscopic images. Dorsal surfaces of irradiated lungs from representative animals at 12 months post-irradiation. (BB = broad beam, MB = microbeams. Spatial values represent the widths of microbeams. Dose values represent peak entrance doses. White bar = 5 mm)

Images of the dorsal surface of irradiated lungs from representative animals are shown in Figure 3. Lesions are easily seen in animals irradiated with broad beams at entrance doses of both 30 (shown) and 50 Gy (not shown). No lesions are seen in animals exposed to 50$\mu$m wide microbeams at peak entrance doses of 300 Gy and lower, while small, patchy lesions are seen at the highest dose of 600 Gy. Of the animals irradiated with 500$\mu$m wide microbeams, no lesions are apparent in those exposed to peak entrance doses of 50 and 100 Gy, but scarring is observed at the higher doses of 300 and 600 Gy.

Fibrosis

Table 3 presents the highest fibrosis scores observed for each animal in the study. The fibrosis scoring broadly follows the results of the macroscopic imaging presented in Figure 3. Significant fibrotic damage occurs for animals irradiated with broad beams at both 30 and 50 Gy, exhibiting scores of 5. For animals irradiated with 50$\mu$m wide microbeams, fibrosis scores are very low for doses up to 300 Gy, with values ranging between 0 and two. At 600 Gy, the fibrosis scores are slightly higher, but still mostly at a value of two. For animals irradiated with 500$\mu$m wide microbeams, fibrosis scores are again very low, at a value of one, for doses of 50 and 100 Gy. For doses of 300 and 600 Gy, however, significant fibrotic damage is present with scores of four to five.

**Table 3:** Fibrosis scores. Highest fibrosis scores observed at 12 months post-irradiation.

Irradiation Group	Dose (Gy)	Animal ID	Fibrosis Score
Control	-	α-1	0
		α-2	0
		-	-
		-	-
Broad beam	30	β-1	4
		β-2	5
		-	-
		-	-
	50	γ-1	5
		γ-2	5
		-	-
		-	-
Microbeams, 50 μm	50	δ-1	0
		δ-2	0
		-	-
		-	-
	100	ε-1	2
		ε-2	1
		ε-3	1
		ε-4	1
	300	η-1	1
		η-2	2
		η-3	1
		η-4	2
	600	θ-1	3
		θ-2	2
		θ-3	2
		θ-4	2
Microbeams, 500 μm	50	κ-1	1
		κ-2	1
		κ-3	1
		-	-
	100	λ-1	1
		λ-2	1
		λ-3	1
		-	-
	300	ρ-1	5
		ρ-2	4
		ρ-3	5
		-	-
	600	σ-1	4
		σ-2	4
		σ-3	4
		σ-4	4

**Figure 4:**
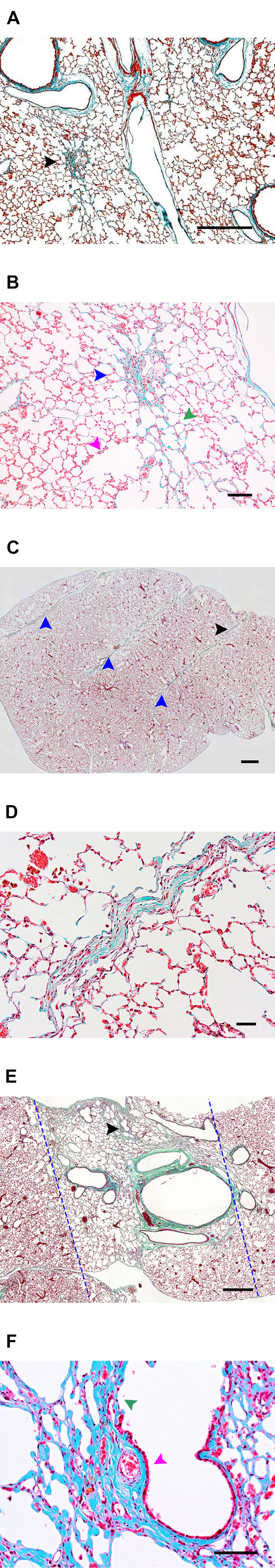
Fibrotic lesions. Fibrotic regions of lung samples were taken from representative animals at 12 months post-irradiation. (A) Animal $\theta$-1 irradiated with 50$\mu$m wide microbeams on a 400$\mu$m pitch with a peak entrance dose of 600 Gy. The black arrow points to a region of low-grade perivascular fibrosis shown magnified in (B). Black bar = 500$\mu$m. (B) The blue arrow points to a fibrotic lesion with a score of 3. The green arrow points to a region of limited, small fibrotic interalveolar septa. The magenta arrow points to dilated alveoli. Black bar = 100 $\mu$m. (C) Large area sample from animal $\sigma$-1 irradiated with 500$\mu$m wide microbeams on a 4 mm pitch with a peak entrance dose of 600 Gy. The blue arrows point to dense high-grade fibrotic stripes marking the beam paths. The region indicated by the black arrow is magnified in (D). Black bar = 1 mm. (D) Detail of thick fibrotic stripe with a score of 4. A few collapsed alveoli are integrated into the fibrotic stripe. A few hemosiderin laden macrophages are present (brown pigment). Black bar = 500$\mu$m. (E) Animal $\gamma$-2 irradiated with a broad beam radiation field with an entrance dose of 50 Gy. A broad fibrotic lane roughly demarcated by the blue dashed lines replaces normal lung structure. The black arrow indicates a region shown magnified in (F). Black bar = 500$\mu$m. (F) Massive fibrosis of score 5 is observed, along with loss of bronchiolar/alveolar epithelia, and alveolar collapse and induration. The magenta arrow points to preserved bronchial epithelium lining, stained red.  The green arrow points to missing bronchial epithelium lining, replaced with hypocellular, compact fibrous tissue, stained green. Black bar = 100$\mu$m.

Representative microscopic images of lung tissues exposed to the highest doses are shown in Figure 4. Connective tissues, including fibrotic lesions, are colored green from the Masson-Trichrome stain. 50$\mu$m wide microbeams on a 400$\mu$m pitch with a peak entrance dose of 600 Gy (Figure 4AB) produce negligible, inconsistent, and patchy fibrosis. The paths of individual microbeams cannot be seen. Conversely, 500$\mu$m wide microbeams on a 4 mm pitch at the same entrance dose (Figure 4CD) produce dense fibrotic stripes marking the beam paths. At various points along those paths, destruction and obliteration of airways and vasculature is seen. From the seamless broad beam radiation field with an entrance dose of 50 Gy (Figure 4EF), a dense fibrotic lane within the beam path is observed with extensive alveolar collapse and induration.

**Figure 5:**
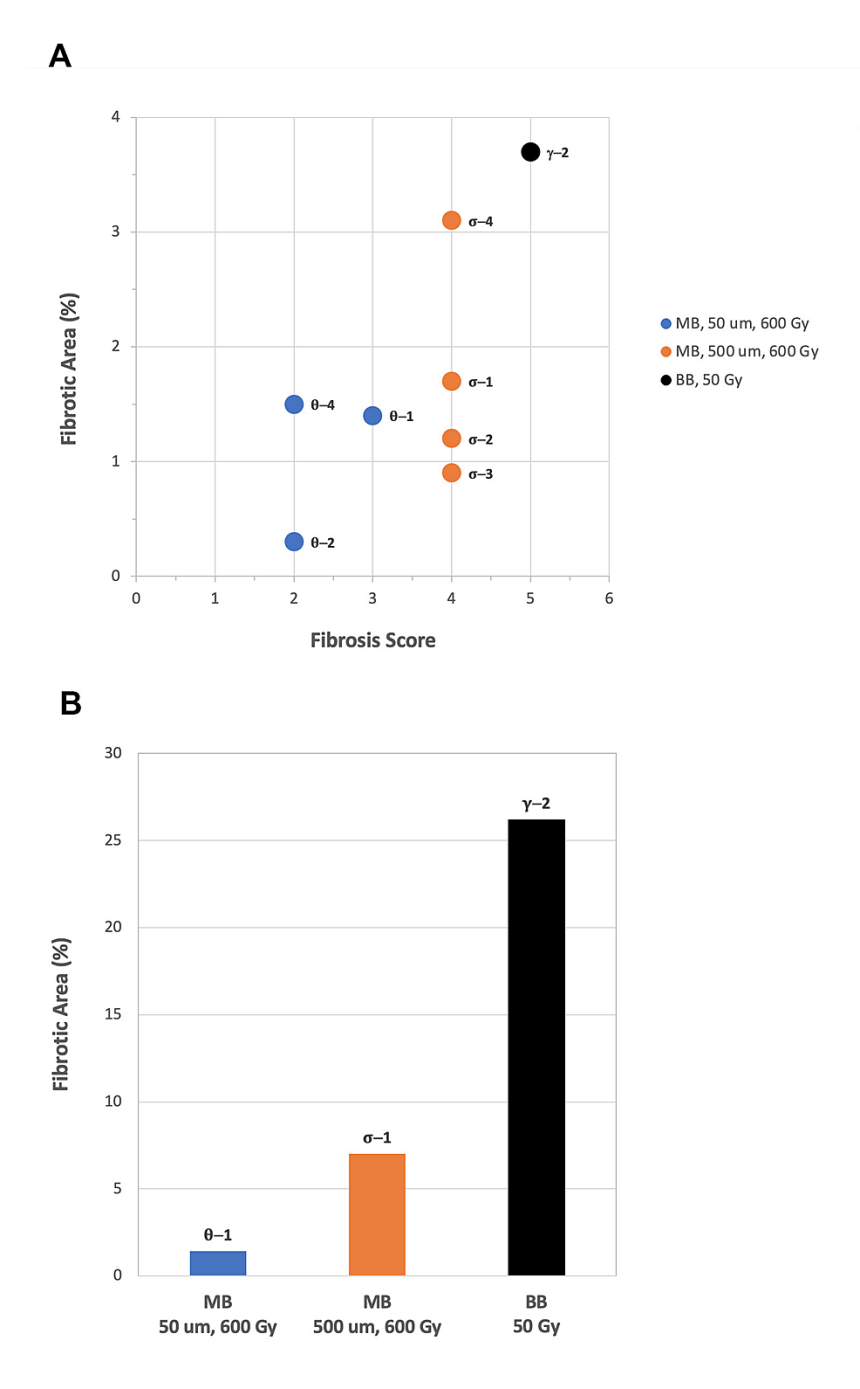
Fibrotic extent. Measurements of fibrotic lesion extent in selected animals. (A) The fibrotic area, as a percentage of the total radiation window area (1 cm^2^), associated with the highest fibrosis score observed in the specified animal. (B)The sum of all fibrotic lesion areas with a score $\geq$ 3 in the specified animal. (BB = broad beam, MB = microbeams. Spatial length values represent microbeam widths. Dose values represents peak entrance doses. Animal IDs are adjacent to the data points in (A) and above the bars in (B).)

Measurements of the areas occupied by fibrotic lesions were made on eight animals with typical findings among the highest dose groups. Figure 5A shows a plot of fibrotic area versus fibrosis score for these eight animals. The fibrotic area is presented as a percentage of the total radiation window area (1 cm^2^). Figure 5B shows the sum of fibrotic areas with fibrosis scores $\geq$ 3 for three representative animals. It is apparent that microbeams induce much less fibrotic damage than broad beams.

Inflammation

Acute and chronic inflammatory changes in terminal bronchioles, alveoli, and pleura (i.e., bronchiolitis, pneumonitis, pneumonia, pleuritis), either in an alveolar and interstitial or bronchioloalveolar pattern, are unobtrusive compared to the fibrotic lesions. Inflammatory changes are more generally present in the broad beam group, occasionally in animals exposed to 500 $\mu$m wide microbeams, and least appreciable in animals irradiated with 50 $\mu$m wide microbeams. Mast cells are occasionally noted in fibrotic areas. Alveolar macrophages with foamy cytoplasm, probably lipid-laden, are correlates of macroscopic yellow spots shown in Figure 3 for the case of 50 $\mu$m wide microbeams with a peak entrance dose of 600 Gy. Isolated cholesterol granulomas are also observed. Alveolar mucinosis occurs near partially destroyed bronchi in some animals irradiated with 500 $\mu$m wide microbeams.

Bronchial damage

Among the animals irradiated with 500 $\mu$m wide microbeams, 50% of those exposed to 300 and 600 Gy peak entrance doses display bronchial lesions located within the paths of the peak zones. The lesions consist of the destruction of bronchial epithelium and other bronchial wall components, resulting in a partial or total loss of the walls of small or medium-sized bronchi, and their partial or total obliteration by hypocellular, often hyaline fibrous tissue. See Figure 4F. Animals exposed to 500 $\mu$m wide microbeams with 50 and 100 Gy peak entrance doses, and animals exposed to 50$\mu$m wide microbeams at all doses, do not exhibit bronchial lesions.

Vascular damage

The most conspicuous changes in larger blood vessels are the thinning, the complete loss, or the fibrous obliteration of the tunica media of bronchial and pulmonary arteries in the path of 500 $\mu$m wide microbeams with peak entrance doses of 300 and 600 Gy.

Only isolated, few, small extravasations of erythrocytes into alveoli are noted in the lungs of all three irradiated groups. Hemosiderin granules in macrophages in alveoli, interstitium, tunica propria of bronchi and pleura also occur in all groups, with a small preponderance along the irradiation path in the broad beam group, followed by the 500$\mu$m wide microbeam group, and lastly by the 50$\mu$m wide microbeam group. These hemosiderin-laden macrophages sometimes appear along with streaks in the 500$\mu$m wide microbeam group, while appearing scattered in the 50$\mu$m wide microbeam group.

Weight

All animals gained weight over the duration of the study, starting at approximately 300 g and ending at approximately 500 g. The animals in the control and 50$\mu$m microbeam groups gained the most weight, whereas the animals in the 500$\mu$m microbeam and broad beam groups gained the least. At the end of the study, the control group weighed approximately 20% more than the lowest weight-gain group.

## Discussion

Of the three radiation field patterns employed in this work, microbeams 50$\mu$m wide on a 400$\mu$m pitch displayed the greatest normal tissue tolerance. The grade and extent of fibrotic lesions, inflammation, bronchial and vascular damage were remarkably low at even the highest peak entrance dose of 600 Gy.

Microbeams 500$\mu$m wide on a 4 mm pitch also exhibited high normal tissue tolerance at peak entrance doses ≤ 100 Gy. At doses ≥ 300 Gy, however, fibrosis and bronchial and vascular damage were severe and extensive.

Both FLASH-MRT field patterns fared far better than the FLASH-RT pattern. At a dose of 50 Gy, broad beam radiation-induced measurable fibrotic damage over 25% of the irradiated area whereas 50$\mu$m microbeams produced none and 500$\mu$m microbeams generated negligible fibrotic damage.

At a dose of 30 Gy, broad beam radiation produced high-grade fibrotic damage. This result is consistent with the results of Favaudon et al*.* [10]. With 50$\mu$m microbeams, however, a peak entrance dose of 300 Gy produced only low-grade damage. Thus, FLASH-MRT provides at least an order of magnitude improvement in normal lung tissue radio-resistance compared to FLASH-RT. 

Although the focus of this study is on the late-stage injury of radiation-induced fibrosis, it is noted that signs of significant inflammation associated with the early-stage injury of radiation-induced pneumonitis were minimal for 50$\mu$m microbeams at all doses and for 500$\mu$m microbeams at doses ≤ 100 Gy. While it is possible that inflammation may have been more prominent in these cases at earlier times, it had largely resolved by the time point of 12 months post-irradiation.

While this work does not include a direct comparison of FLASH-MRT or FLASH-RT to CONV-RT, the study of Favaudon et al. [10] demonstrated that FLASH-RT is superior to single fraction CONV-RT in providing normal lung tissue radio-resistance. A study by Prezado et al. [35] compares conventional dose rate microbeam radiotherapy (CONV-MRT) to CONV-RT applied to the normal brain tissue of rats. The study employed 700$\mu$m microbeams on a 1.5 mm pitch. A CONV-RT single-fraction dose of 20 Gy produced severe tissue damage, while a peak dose of 58 Gy in the CONV-MRT microbeam array produced no damage. Thus, at both conventional and FLASH dose rates, microbeams provide superior normal tissue sparing.

Traditionally, doses of 30 and 50 Gy applied in a single fraction, as employed in this study, would be considered stunningly large in the context of CONV-RT. However, a recent Phase II clinical trial of stereotactic body radiation therapy for inoperable early-stage lung cancer found a 34 Gy single fraction treatment protocol to be effective. The protocol-specified endpoint for the trial was the rate of adverse (toxicity) events. By far, the greatest number of adverse events were respiratory disorders. 51% of patients displayed such events. 23% displayed radiological evidence of fibrosis [36]. The findings of this study suggest that a FLASH-MRT treatment protocol would yield a much lower rate of adverse events.

The peak microbeam doses of 100 Gy and higher utilized in this study are expected to be of therapeutic value upon application to lung and other tumors. FLASH-MRT has been shown to control murine mammary carcinomas at peak doses as low as 75 Gy [37].

The biophysical and biochemical processes invoked by the spatial fractionation component of FLASH-MRT which yield improved normal tissue radio-resistance are complex and not entirely understood. However, it has been hypothesized that the following general sequence of events occurs [38]: first, the irradiated cells generate signals to the adjacent nonirradiated cells indicating the presence of damage; these signals may include the release of cytokines or cell-to-cell gap-junction communication; second, the nonirradiated cells respond with repair effects; these effects may include stimulation of the immune system to remove apoptotic cells in the damaged region, the release of growth factor proteins leading to enhanced normal cell proliferation, and migration of the normal cells into the damaged region. Because the regions of tissue damaged by the microbeams are so thin, the above processes are effective in the entire wound volume and constitute effective healing. Damage to normal tissue with microbeams occurs when the microbeams are too wide, or when radiation scattering events from the microbeams yield doses in the spaces between the beams which are too high to be tolerated. In the latter instance, all normal tissue has received too much radiation and a healing response is not possible.

Much experimental data indicate that the preferential tumoricidal effect of FLASH-MRT is the result of a difference in the radio-vulnerabilities of normal and tumoral tissue microvasculatures [18, 37, 39-42] (see also Abstract: Dilmanian FA, Hainfeld JF, Kruse CA, et al.: Biological Mechanisms Underlying the Preferential Destruction of Gliomas by X-ray Microbeam Radiation. National Synchrotron Light Source Activity Report. US Government Printing Office, Washington, DC; 2002.) Normal tissue microvasculature is comprised of a high density of small, evenly distributed, well-differentiated arteries, veins, and capillaries. Tumoral tissue microvasculature, however, is characterized by a lower density of vessels which tend to be larger and chaotic in distribution and function. Blood flow may proceed in alternating directions in the same vessel. So-called “mother vessels” in tumoral tissue have degraded basement membranes and pericyte detachment [43]. Upon exposure to FLASH-MRT, the microvasculature of normal tissue tends to heal, whereas the tumoral microvasculature is disrupted and becomes highly permeable, leading to death by ischemia.

The most prominent hypothesis for improved normal tissue radio-resistance arising from the temporal characteristics of FLASH radiation is transient, profound, radiation-induced hypoxia [44]. Favaudon et al. [10] discounted this hypothesis primarily because their study showed that CONV-RT and FLASH-RT provided equivalent tumor control. Conventional wisdom suggests that radiation-induced hypoxia should not only lead to increased radioresistance for normal tissue, but also for tumoral tissue. However, in a recent paper by Spitz et al. [45], it is postulated that differences in the radiochemistry dynamics at FLASH timeframes within normal and tumoral tissues account for the greater cell killing in tumoral tissue. Specifically, tumor cells have as much as four times more labile iron (Fe+2) than normal cells. Fenton-type reactions with labile iron significantly increase reactive oxygen species chain reactions and thus produce more aggressive killing of cancer cells than normal cells. The greater number of Fenton-type reactions also suggests that the rate of clearing of peroxyl radicals and hydroperoxides is significantly lower in tumoral tissue compared to normal tissue after FLASH irradiation, thereby allowing more time for cell killing in cancerous tissue than in normal tissue.

This study is a gateway study, designed to determine whether or not an investigation of the effect of FLASH-MRT on lung tumors is warranted. The positive results suggest so.

There are problems impeding the arrival of FLASH-MRT into the clinic [46]. Foremost among these is the fact that microbeams with sufficiently low beam divergence and sufficiently high dose rate can currently only be produced by third-generation synchrotrons. Synchrotrons are very large devices that only nation-states can afford. Recently, however, a few groups throughout the world have begun developing a new type of radiation source which employs inverse Compton scattering (ICS) [47-49]. An ICS source has the potential to rival synchrotrons for FLASH-MRT with a tractable footprint and cost. These devices will also be able to produce higher energy photons more easily, allowing for improved radiation depth-dose profiles. These are promising developments.

A second issue that has limited the interest in FLASH-MRT among radiotherapy equipment manufacturers is use case. Nearly all FLASH-MRT studies to date have focused on the central nervous system. Such cancers account for approximately 2% of all cancers [2]. Demonstration that FLASH-MRT can service many types of cancer would help to justify the large investment necessary to develop a clinical product. The results of this study begin to break down that barrier, showing that FLASH-MRT circumvents the largest impediment to the treatment of lung cancer with radiotherapy--normal tissue toxicity.

## Conclusions

FLASH-RT is currently of great interest in the radiotherapy community due to demonstrations of increased normal tissue radio-resistance in lung and other anatomy. Our work demonstrates that FLASH-MRT provides an order of magnitude improvement in normal tissue radio-resistance in lung anatomy compared to FLASH-RT. This result opens the door to investigations of the efficacy of FLASH-MRT against lung cancer, a deadly disease of a vast proportion.

In addition, we have noted in this article some of the barriers delaying the arrival of FLASH-MRT into the clinic. We have highlighted the promising development of ICS radiation sources, which has the potential to overcome the most significant of these barriers.
